# O2-2 Should I stay or should I go? A mixed-methods, longitudinal investigation of predictors, barriers and enablers of adherence to REACT, a 12-month group-based, active-ageing programme

**DOI:** 10.1093/eurpub/ckac094.010

**Published:** 2022-08-29

**Authors:** Janet Withall, Jolanthe de Koning, Rosina Cross, Kate Sutton, Colin Greaves, Jess Bollen, Sarah Moorlock, Linda Lee, James Clynes, Afroditi Stathi

**Affiliations:** 1 Department for Health, University of Bath, Bath, United Kingdom; 2 School of Sport, Exercise and Rehabilitation Sciences, University of Birmingham, Birmingham, United Kingdom; 3 College of Medicine and Health, University of Exeter, Exeter, United Kingdom

**Keywords:** Physical activity, older adults, physical function, randomised control trial, adherence

## Abstract

**Background:**

Physical activity (PA) programmes targeting older adults often report relatively low attendance rates which limits impact. Research into barriers and enablers of PA adherence is often qualitative and rarely tests outcomes against objectively monitored adherence to assess whether what people say is actually reflected in what they do. This study adopts a rare, mixed methods, longitudinal perspective identifying subjective and objective predictors of and associations with adherence to REtirement in ACTion (REACT), a 12-month physical activity intervention for frail or pre-frail older adults.

**Methods:**

Semi-structured interviews conducted at six (n = 17) and 12 months (n = 10) explored barriers and enablers to adherence. Thematic analysis led to ten adherence related research hypotheses. These were tested by examining correlations between REACT programme attendance and physical function (Short Physical Performance Battery), self-rated physical function (mobility assessment tool-short form (MAT-sf)), dominant hand grip strength assessed by digital dynamometer, Ageing Well profile (social scale), process evaluation data at baseline (n = 411) and six-months (n = 348) and open-ended participant feedback at six-months (n = 307). Each participant response was scored -1) for a negative comment, 1 for positive or zero for no comment or balancing negative and positive comments.

**Results:**

Higher adherence correlated with younger age (r=-0.162, p > 0.001), better physical function, both objectively measured (r = 0.118, p > 0.05) and self-rated (r = 0.134, p > 0.01), greater grip strength (r = 0.118, p > 0.05) and having less social contact (r=-0.134, p > 0.01), at baseline. It also correlated with an improvement in objectively measured physical function between baseline and six months (r = 0.200, p > 0.001). At 6-months enjoyment of the programme (r = 0.263, p > 0.001), specifically enjoyment of muscle-strengthening exercises (r = 0.142, p > 0.027), perception of positive social interactions (from questionnaire data (r = 0.212, p > 0.001) and open-ended feedback (r = 0.157, p > 0.01)) and perceptions of an autonomy-supportive teaching style (r = 0.213, p > 0.001) all correlated with higher adherence.

**Conclusions:**

PA programmes for older adults should encourage the development of social connections and group cohesion but should take a flexible approach to avoid negatively affecting adherence amongst those with pre-existing high levels of social contact. Building confidence in PA and physical function as these improve during the programme, promoting enjoyment and utilising an autonomy-supportive leader teaching style are important in order to support adherence.

